# Academic inequality through the lens of community ecology: a meta-analysis

**DOI:** 10.7717/peerj.1457

**Published:** 2015-12-03

**Authors:** Akira S. Mori, Shenhua Qian, Shinichi Tatsumi

**Affiliations:** 1 Graduate School of Environment and Information Sciences, Yokohama National University, Yokohama, Japan; 2 Postdoctoral Station of Ecology Chongqing University, Chongqing, China

**Keywords:** Publication bias, Dominance, Societal hierarchy, Meta-analysis, Species abundance distributions

## Abstract

Ecological assemblages are generally characterized by a few dominant species and numerous others. Such unequal distributions of dominance also emerge in human society, including in scientific communities. Here, based on formal community ecological analyses, we show the temporal trends in the number of scientific publication in the discipline of “ecology.” Based on this, we infer possible factors causing the imbalance of reputation and dominance among countries. We relied on 454 ecological meta-analysis papers published from 1998 to 2014, which sourced over 29,000 original publications. Formal meta-analyses are essential for synthesizing findings from individual studies and are critical for assessing issues and informing policy. We found that, despite the rapid expansion of outlets for ecology papers (analogous to an increase in carrying capacity, in ecological systems), country diversity as determined from first author affiliations (analogous to species diversity) did not increase. Furthermore, a country identity was more powerful than the popularity of the scientific topic and affected the chance of publication in high-profile journals, independent of the potential novelty of findings and arguments of the papers, suggesting possible academic injustice. Consequently, a rank order and hierarchy has been gradually formed among countries. Notably, this country-dominance rank is not only specific to this scientific domain but also universal across different societal situations including sports and economics, further emphasizing that inequality and hierarchical structure exist even in modern human society. Our study demonstrates a need for having robust frameworks to facilitate equality and diversity in the scientific domain in order to better inform society and policy.

## Introduction

Inequality is ubiquitous in nature. In natural ecosystems, biological assemblages are fundamentally characterized by a skewed distribution of abundance, with a few major (abundant) species and numerous minor (rare) species (Magurran & Henderson, 2003; McGill et al., 2007). This unequal distribution of dominance among species can be recognized from a skewed shape of species-abundance distributions (SADs). To understand the mechanisms of inequality and diversity in species assemblages, community ecologists have relied on SADs (Magurran & Henderson, 2003; Magurran & McGill, 2011; McGill et al., 2007; Nekola & Brown, 2007; Simons et al., 2014) since the 1930s (Doi & Mori, 2013). Notably, such inequality is not only observed in natural systems but is also pervasive in many systems and situations including modern human society (Deaton, 2014; Mechanic, 2002; Ravallion, 2014). Distributions of personal income, stock volumes for corporations, and even the citation frequency of scientific papers often follow patterns analogous to SADs (Nekola & Brown, 2007). As explicit from the last example, inequity is also inherent in the world of science for many reasons (Budden et al., 2008; Clavero, 2010; Henrissat, 1991; Leimu & Koricheva, 2005). The remarkable similarity between ecological and human communities, in terms of SAD-like patterns, implies that the possibility of gaining insight into the causes and consequences of academic inequality (or dominance hierarchy) through the lens of community ecology. In other words, a series of community ecology analyses, including the assessment of SADs, could be useful to evaluate the dominance, evenness and diversity of scientific communities.

In doing so, this study focuses on scientific communities in the discipline of ecology. Note that inequality in ecological assemblages does not come with any moral associations as long as they are structured under natural (not anthropogenic) drivers. We thus emphasize that our primary aim is to evaluate spatial and temporal patterns of potential academic inequality, and not to give any moral connotations that impart a natural justification for human inequality. With this in mind, this study focused on scientific communities in the discipline of ecology. Specifically, we relied on ecology papers based on a meta-analysis (hereafter, meta-analysis papers).

We have several rationales for considering that inequality in meta-analytical publications could potentially have large consequences. Quantitative reviews in the form of formal meta-analyses are powerful and essential tools for gaining insight from individual research papers (Jeffery et al., 2014); they can be a determinant of the future direction of a given field of science, and, owing to these characteristics, they also play a critical role in assessing issues and informing policy (Boyd, 2013; Button & Nijkamp, 1997; Koricheva, Gurevitch & Gómez-Aparicio, 2014). Currently, numerous scholars in ecology and related disciplines are working on meta-analytical assessments to fulfil the requirements of several global initiatives (Perrings et al., 2011b); this new assessment body is the Intergovernmental Science–Policy Platform on Biodiversity and Ecosystem Services (IPBES) (Perrings et al., 2011a; Turnhout et al., 2012), which is open to all member countries of the United Nations. Although this exemplification may be the one extreme and not all meta-analyses aim to be policy-relevant, it is highly likely that meta-analysis papers, especially when published in high-profile journals, have large impacts on society that can disperse beyond the discipline.

## Methods

### Data collection

We assembled a representative sample of meta-analysis papers in ecology as follows. We searched the literature using the ISI Web of Science database, using a combination of “ecology” and “meta-analysis” as keywords (for topics). This literature search matched 456 publications (as of March 1st, 2015). We reviewed the literature to find publications that fit within the topic of ecology. Note that some papers used the term “meta-analysis” in an inappropriate way (Koricheva, Gurevitch & Gómez-Aparicio, 2014). To synthesize the results from different studies with different spatial and temporal scales, different sample sizes, and potential publication biases, a meta-analysis should be conducted with appropriate statistics. Therefore, we focused on those manuscripts based on the calculation of statistics, considering variations among original data (e.g., effect sizes); we excluded studies based on qualitative approaches (e.g., review) or other inadequate quantification (e.g., vote-counting) (Koricheva, Gurevitch & Gómez-Aparicio, 2014). We also excluded those partially based on original data and thus had different approaches for the calculation of statistics. Prior to 1998, the number of papers were sporadic (often no meta-analysis papers). Accordingly, we focused on the period of 1998–2014. This screening resulted in 171 publications. Then, to ensure our coverage of data, we visited publication websites of journals categorized in ecology in the ISI Web of Science, which had at least two ecological meta-analysis papers from the initial screening, and used the same keywords to search for additional literature. Some multidisciplinary journals occasionally publish ecology papers, so we also visited the websites of these journals (Nature, Nature Communications, Science, Proceedings of the National Academy of Sciences, and Annals of the New York Academy of Sciences), using the same keywords to find additional literature. As a result, we found an additional 448 publications, which we screened using the same procedures described above, resulting in 283 publications.

We combined our data obtained through the above screenings and this resulted in a total of 454 formal meta-analysis publications in the discipline of ecology (Table S1). For these papers, we recorded (1) publication year; (2) journal; (3) first author’s affiliated country (if multiple addresses are listed, we used the affiliation shown first); (4) sample sizes; (5) number of original publications; (6) study categories; and (7) journal impact factor (IF) from the ISI Journal Citation Reports. For sample sizes, we examined the literature and recorded sample sizes (e.g., number of comparisons) for their calculations of effect sizes and other valid meta-analytical statistics. Some papers had multiple comparisons for different topics within a single meta-analysis paper, so we summed sample sizes by carefully reading through them and excluding duplications. The study categories were classified as follows: (6-1) study taxa (plant, vertebrate, invertebrate, microbe, or multiple taxa), (6-2) practical issue (meta-analysis oriented to applied issues such as biological conservation and ecosystem restoration, or no explicit focus on such applied issues), (6-3) study system (terrestrial system, freshwater system, marine system, or combination of these), and (6-4) manipulation (manipulated system, unmanipulated (natural) system, theoretical (simulated) system, or combination of these). For the IF, we assigned the IF from the preceding year to the publication year of each meta-analysis paper (e.g., a paper published in 2005 was assigned to 2004 IF). Note that the 2004 IF is already a year behind, since it is based on 2004 citations to articles published in 2002–2003 and thus reported in 2005. We assume that this is reasonable, since authors might use the 2004 IF to decide where to submit their paper in the year of 2005. A checklist and diagram for our paper selection are provided in Table S2 and Fig. S1.

### Data analyses

In community ecology, one of the basic formats for data analyses is to have matrix data with species in columns and sites in rows. Each cell of the matrix often has the observed number of individuals, coverage, biomass, or binary record of presence/absence. We converted our data to a matrix of *journal* (column) × *year* (row), first author’s *country* affiliation × *year* and first author’s *country* affiliation × *journal*. We primarily used these three matrices for community analyses. Each cell contained the number of meta-analysis papers (corresponding to abundance in ecological communities). We used R software 3.0.2 (R Core Team, 2013) for all analyses using “vegan” (Oksanen et al., 2013), “lme4” (Bates et al., 2014), and “ppcor” (Kim, 2012) libraries.

**Figure 1:**
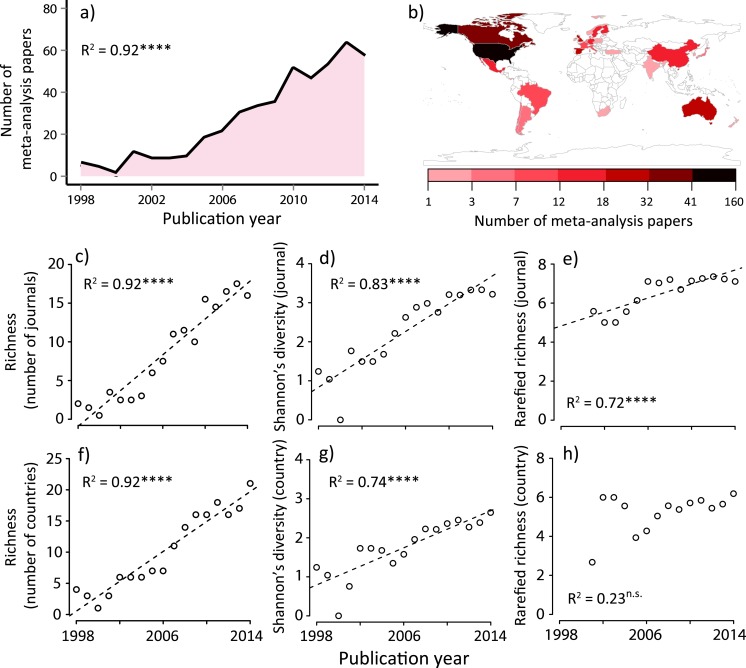
Summary of meta-analysis papers in ecology; (A) temporal trend to date, (B) spatial distribution to date, temporal changes in (C) journal richness, (D) Shannon’s diversity for journals, (E) rarefied journal richness, (F) country richness, (G) Shannon’s diversity for countries, and (H) rarefied country richness. Country is based on the first author’s country affiliation. The *R*^2^ values are based on ordinary least-square regressions. Significant levels; ^∗∗∗^*P* < 0.001 and n.s. *P* > 0.05. The map was generated using R software 3.0.2 (http://www.r-project.org/).

Analogous to species diversity calculations, we calculated richness (total number) and Shannon’s diversity index (accounting for number and abundance) for the journal and country diversity in each year. Shannon’s diversity index (*H*) is defined as $H=-\sum _{i=1}^{S}({p}_{i}\ln \hspace{0.167em} {p}_{i})$, where the proportion of species *i* relative to the total number of species (*p_i_*) is calculated, and then multiplied by the natural logarithm of this proportion (ln *p_i_*) (Magurran & McGill, 2011). Note that species *i* should be treated as journal *i* or country *i* in this study. As diversity estimations are often affected by a sampling effort, we relied on an individual-based rarefaction (Gotelli & Colwell, 2001). This correction was used to account for the sampling effect, in which larger samples have a higher probability of including more species. In our study, given the significant increase in the number of publications in the ecology discipline (Fig. S2) and that of ecological meta-analysis papers (Fig. 1A), this correction is important. We first calculated the number of papers expected for each year based on a rarefaction curve. We found that the individual years similarly fit one common rarefaction curve (Fig. S3A), suggesting that the differences in diversity among years were attributable to the number of papers published in different years. This is equivalent to more individual effect (Šímová et al., 2011), which can emerge as a result of the sampling effect. In ecological communities, since a greater number of individuals can be divided into more species, resource-rich habitats which can support more individuals will then support more species (Šímová et al., 2011). To correct for this sampling bias, we calculated the richness values for the number of 8 meta-analysis papers in each year. Additionally, to determine per-capita diversity values, we divided observed richness estimates by the total number of meta-analysis papers in each year. This correction is to remove the effects of the temporal increases in total number of meta-analysis papers (Fig. 1A), which is analogous to an increase in carrying capacity in ecological systems.

We conducted an additional verification to determine that our dataset was robust enough to conduct additional community analyses and to check for possible undersampling in our dataset. We relied on an extrapolation technique based on a nonparametric richness estimator (with the second-order jackknife estimator), as has been previously reported (Laliberte, Zemunik & Turner, 2014). We assessed the reliability of our dataset by using a sample-based rarefaction curve for the *country* × *year* matrix, with the curve depicting the average of 999 random permutations (Fig. S3B). This analysis showed that total country richness was saturating, indicating that our dataset spanning the focal period of 1998–2014 reasonably included almost all potential candidates of author country affiliations for ecological meta-analysis papers.

We assessed the unequal distribution of abundance among first author country affiliations using species abundance distributions (SADs). Although SADs may not be neccesarily a perfect tool (Adler, Hillerislambers & Levine, 2007), their ability to define dominance and rarity in a particular system are increasingly becoming important to give theoretical and practical implications for understanding the underlying mechanisms of community organization (Magurran & McGill, 2011; Matthews, Borges & Whittaker, 2014; McGill, 2010; McGill et al., 2007; Simons et al., 2014). The SADs can be plotted differently using Whittaker or Preston plots. Following a recent meta-analysis on the SADs (Ulrich, Ollik & Ugland, 2010), we used the Whittaker plot. We used the *country* × *year* matrix to test for possible inequality and dominance rank among different countries as our primary focus. We first fit our data to several SADs (brokenstick, pre-emption, log-normal, Zipf and Zipf-Mandelbrot models available in “vegan” library of R software), and compared the fitness based on the Akaike Information Criterion (AIC). As a result, we employed the log-normal model for our data. Reflecting the above results of the richness estimator, we primarily used the dataset containing all focal years of 1998–2014. We conducted the same calculations of log-normal SAD for the data until 2005 and 2010 to determine the temporal fluctuation in dominance-rank among first author country affiliations. Additionally, we calculated the SADs for each 5-year window from 1998 to 2012.

We also attempted to disentangle factors affecting the IFs of each meta-analysis paper. Although the IF is not an absolute measure, it is one of the common metrics for describing the relative profile of scientific journals. Additionally, a previous study on the topic of publication bias in ecology (e.g., due to gender inequality, linguistic injustice, and status of individual scientists) relied on this metric (Clavero, 2011). The IF is most likely an important determinant for citation frequency of papers in many disciplines including ecology (Leimu & Koricheva, 2005). We used linear mixed effect models to account for nested complexity in our dataset. We used the publication year as a random term because the IF changes through time (generally increasing) such that earlier papers tended to have lower IFs. We used first author’s country affiliation, sample sizes, number of original publications, and four study categories (Fig. S4) as potential explanatory variables. We constructed more than 60 models with different explanatory variables with random slope, random intercept, or both random slope and intercept. Using the lowest AIC values among the candidate models, we determined the best model that had all variables (i.e., first author’s country affiliation, sample sizes, number of original publications, and four study categories) with a random intercept. To see how these explanatory variables had different impacts on the IFs, we excluded one of these variables from the best model, and checked for an increase in the AIC value. Additionally, we fit the data to a plot to predict the IFs based on sample sizes and the number of publications for visual interpretations (we estimated mean values and 95% confidence intervals).

To further determine whether the dominance rank order among countries was robust, we relied on a nestedness analysis. A nested composition pattern in ecological communities emerges when hospitable habitats favor more species, including both dominant and rare species, and less-hospitable habitats have only species that are common throughout habitats (Sasaki et al., 2012). In other words, a significantly nested meta-community emerges when habitats with lower species richness tend to harbor proper subsets of those species present in richer habitats (Almeida-Neto et al., 2008). Based on a NODF (nestedness measure based on overlap and decreasing fills) analysis (Almeida-Neto et al., 2008), we confirmed that the countries was nested in either case, where journals and publication years were considered to be habitats (i.e., in the matrices of *country* (column) × *year* (row) and *country* (column) × *journal* (row)). That is, we tested if dominant countries were common throughout journals or publication years while minor countries emerged only in journals or publication years with more meta-analysis papers. We followed the procedure of previous studies (Rader et al., 2014; Sasaki et al., 2012) and randomized the matrix data to check the significance of nestedness with 999 permutations.

Last, we constructed additional SADs using social and economic data sources. This analysis was to see if our observation for inequality among different countries was only specific to the discipline of ecology or ubiquitous in different societal situations. As measures that represent dominance and reputation of each country, we focused on the total number of Nobel laureates (excluding those in peace and literature) up to 2014, the total number of medals for summer Olympic Games until the present (i.e., up to the 2012 London Games), and gross domestic production in 2014 (billion US dollars) for each country. We fit different SADs with these data as described above. Overall, a log-normal SAD outperformed the others, so that we plot these data into this SAD (Fig. 5). Then, we conducted a linear regression to test for a possible correlation between the number of ecological meta-analysis papers with the other statistics. Note that USA was the most dominant country throughout these data, and to exclude its outlier effect, we relied on a jackknife method (Tukey, 1958). Furthermore, we conducted a partial correlation analysis to remove the possible effects of GDP on the correlations, since GDP had a strong positive correlation with these societal statistics as well as the number of meta-analysis papers. This is because economically developed countries might have a higher probability of having more Nobel laureates and Olympic medals as well as more ecology papers. Again, to exclude a possible outlier effect of the USA, we repeated this partial correlation analysis for data without the USA.

## Results and Discussion

We identified 454 papers that conducted formal meta-analyses in ecology from 1998 to 2014 (‘Methods’), which sourced from 29,747 original studies. We found that the number of ecology papers based on a meta-analysis is heterogeneous both temporally and spatially (Figs. 1A and 1B). Along with the total number of publications in this discipline (Fig. S2), the number of meta-analysis papers increased with time (Fig. 1A). Prior to performing formal community ecology analyses, we verified that our data were sufficient for this purpose based on the rarefaction curves (Fig. S3).

**Figure 2:**
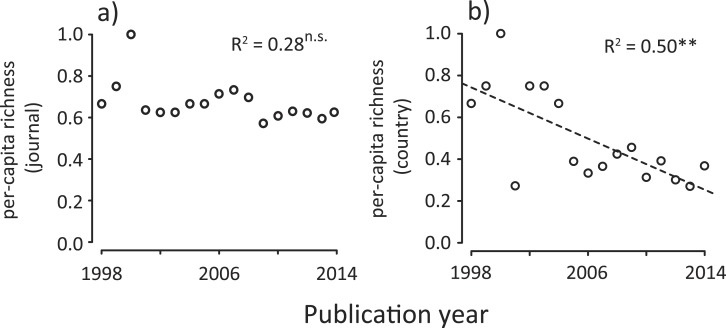
Temporal changes in per-capita country diversity metrics for meta-analysis papers in ecology; (A) journal richness, (B) country richness. Country is based on first author’s country affiliation. The *R*^2^ values are based on ordinary least-square regressions. Significant levels; ^∗∗^*P* < 0.01 and n.s. *P* > 0.05.

We found that the number of journals and that of the first author’s country affiliation (assuming species richness in ecology (Magurran & McGill, 2011)) significantly increased with time (Figs. 1C and 1F). Similarly, Shannon’s diversity indices for journals and countries (corresponding to the species diversity index in ecology, which considers both the abundance of each species and the number of species at the same time (Magurran & McGill, 2011)) significantly increased with time (Figs. 1D and 1G). However, these increases in diversity, in terms of journals and countries, need to be viewed with caution, as they may be a statistical artifact due to the net increase in the number of ecology papers based on a meta-analysis (Fig. 1A). Therefore, we conducted an individual-based rarefaction (Magurran & McGill, 2011) to correct for differences in the number of meta-analysis papers published in different years. We found that, while journal richness significantly increased with time (Fig. 1E), country richness showed a weak temporal increase (Fig. 1H). Furthermore, we standardized diversity indices divided by the total number of meta-analysis papers in each publication year. The results showed that, although there was no change in per-capita journal richness, there was a decreasing trend of per-capita country richness (Fig. 2). These results suggest that, in spite of the rapid expansion of outlets for papers in this scientific domain (analogous to an increase in the number of habitats), country diversity (analogous to species diversity) is not increasing. As a result, there is a tendency that this scientific community is being represented by relatively few dominants.

**Figure 3:**
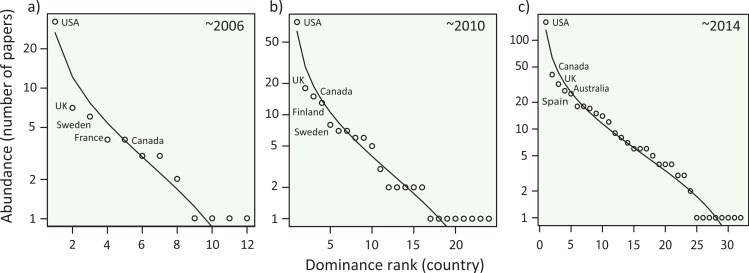
Species abundance distributions (SADs) for meta-analysis papers in ecology. The SADs are plotted with a log-normal model for data (A) between 1998 and 2006, (B) between 1998 and 2010, and (C) between 1998 and 2014. The curves were fit with a log-normal model. Five dominant countries were indicated in each panel. See Fig. S5 for the SADs in each 5-year period.

To determine which country is dominant in the community of ecological science, we plotted the number of meta-analysis papers from each country with a log-normal SAD in a Whittaker plot (‘Methods’), which generally performs well according to a recent meta-analysis on SADs (Ulrich, Ollik & Ugland, 2010). We found that abundance (the number of papers from each country) distribution was left-skewed with a few dominant countries and many rare countries (Fig. 3; Fig. S5), a pattern analogous to biological communities in nature. Although sub-major countries (second to fifth ranked countries) fluctuated through time, the USA continued to outperform others. Notably, three English-speaking countries (the USA, UK and Canada) were constantly dominant, possibly reflecting linguistic injustice in ecological publications (Clavero, 2010), one of the strongest roots of academic inequality (Henrissat, 1991). Because other non-English countries were also found as dominants, factors other than language, such as the number of scientists and budgets for research funding, would affect this country-dominance rank. Although disentangling the underlying mechanisms would not be simple, our results suggest that inequality exists in this scientific community, leading to the decline in per-capita country diversity (Fig. 2). Causal processes for this temporal decline in country diversity may include a cascading effect that the existing inequality further limits the participation of authors from non-dominant countries; a phenomena similar to the competitive exclusion of minor species by dominants that can further skew the SADs of natural species assemblages (Kunte, 2008). We further speculate that other possibilities include the within-paper inequality, which may result from the inequality in the authorships of meta-analyses. That is, if all coauthors would have been included in the analyses country inequalities would blur, but in reality many syntheses (especially at the global scale) tend to include scientists from centrally-located dominant countries as a leading author. All of these mechanisms may generate positive feedbacks, likely contributing to the formation and maintenance of the inequality. Note that, we have gained these speculations from the observed patterns of SADs, but careful interpretation is necessary. In ecological communities, different processes of community organization (e.g., niche versus neutral) can sometimes generate similar patterns of SADs (Adler, Hillerislambers & Levine, 2007). We thus further extended our analysis to determine whether this country-dominance rank was robust, using a nestedness analysis (‘Methods’). One of the reasons for a nested composition pattern in ecological assemblages is a hierarchical ordering of species; i.e., this pattern emerges when hospitable habitats favor more species, including both dominant and rare species, and less-hospitable habitats contain only species common throughout habitats (Rader et al., 2014; Sasaki et al., 2012). The occurrence of nestedness in species assemblages provides clues about processes that affect species distributions and that shape interspecific interactions (Almeida-Neto et al., 2008). Based on the formal analysis (Almeida-Neto et al., 2008), we confirmed that the countries were significantly nested in either case, with journals (NODF = 26.8, *P* < 0.001) and publication years (NODF = 63.4, *P* < 0.001) considered habitats. The finding that countries are configured in a hierarchy, with respect to the number of papers, highlights the presence of academic inequality.

**Figure 4:**
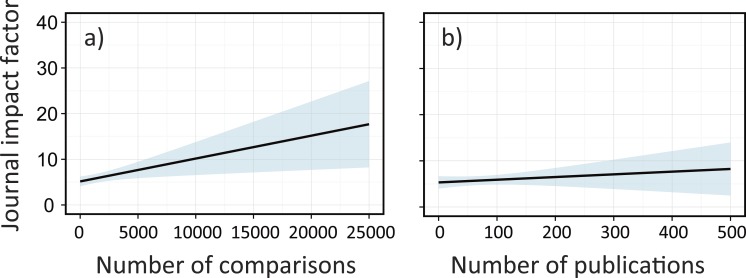
The effects of sample sizes and the number of publications on journal impact factors (IFs) for each meta-analysis paper in ecology; (A) sample sizes and (B) the number of original publications in a single meta-analysis paper. Solid lines are mixed effects models fit across all publication years, which are estimated based on the best model (Table 1). Shaded areas are confidence intervals estimated from the best model (Table 1).

To further evaluate what factors affect academic performance of meta-analysis papers, we used linear mixed effect models that accounted for nested complexity (‘Methods’). Here, we focused on journal impact factors (IFs), which are, although sometimes controversial (Osterloh & Frey, 2015), commonly used as a measure of a journal’s profile (Clavero, 2010) and known to be positively correlated with the citation frequency of papers (Leimu & Koricheva, 2005). We tested various combinations for predictors and found the best model with data sizes (sample sizes and number of original publications), first author’s country affiliation, and study categories (taxa, manipulation, system, and conservation issues) in each paper as explanatory variables and publication year as a random intercept (Table 1). We found that excluding information of sample sizes had the largest impact in reducing the explanatory power of the model, followed by taxa category, manipulation category, country and the number of publications as important predictors (Table 1). IFs significantly increased with sample sizes (Fig. 4A), but showed little change with the number of publications (Fig. 4B). The latter factor generally represents the popularity of a research topic; despite this importance, it was not necessarily linked with a chance of publication in high-profile journals. Notably, the first author’s country affiliation was important in explaining the IFs and was more influential than the amount of research (Table 1), again implying the potential of injustice in this scientific domain. Although the author affiliation does not fully represent the nationality of papers (as many papers include coauthors from different countries), our finding supports similar arguments that have been detailed elsewhere (Leimu & Koricheva, 2005).

**Table 1:** Results of the linear mixed effect models to predict the journal impact factor. The best model, which showed the lowest value of Akaike Information Criterion, included all parameters as an explanatory variable and publication year as a random intercept.

Model	d.f.	AIC
Best model	44	1,206.2
Parameter excluded from the best model		
—Sample sizes	45	1,968.2
—Category (Taxa)	40	1,406.0
—Category (Manipulation)	41	1,305.4
—Country	17	1,284.2
—Number of publications	43	1,237.8
—Category (System)	40	1,221.8
—Category (Applied/basic issues in ecology)	43	1,207.4

**Figure 5:**
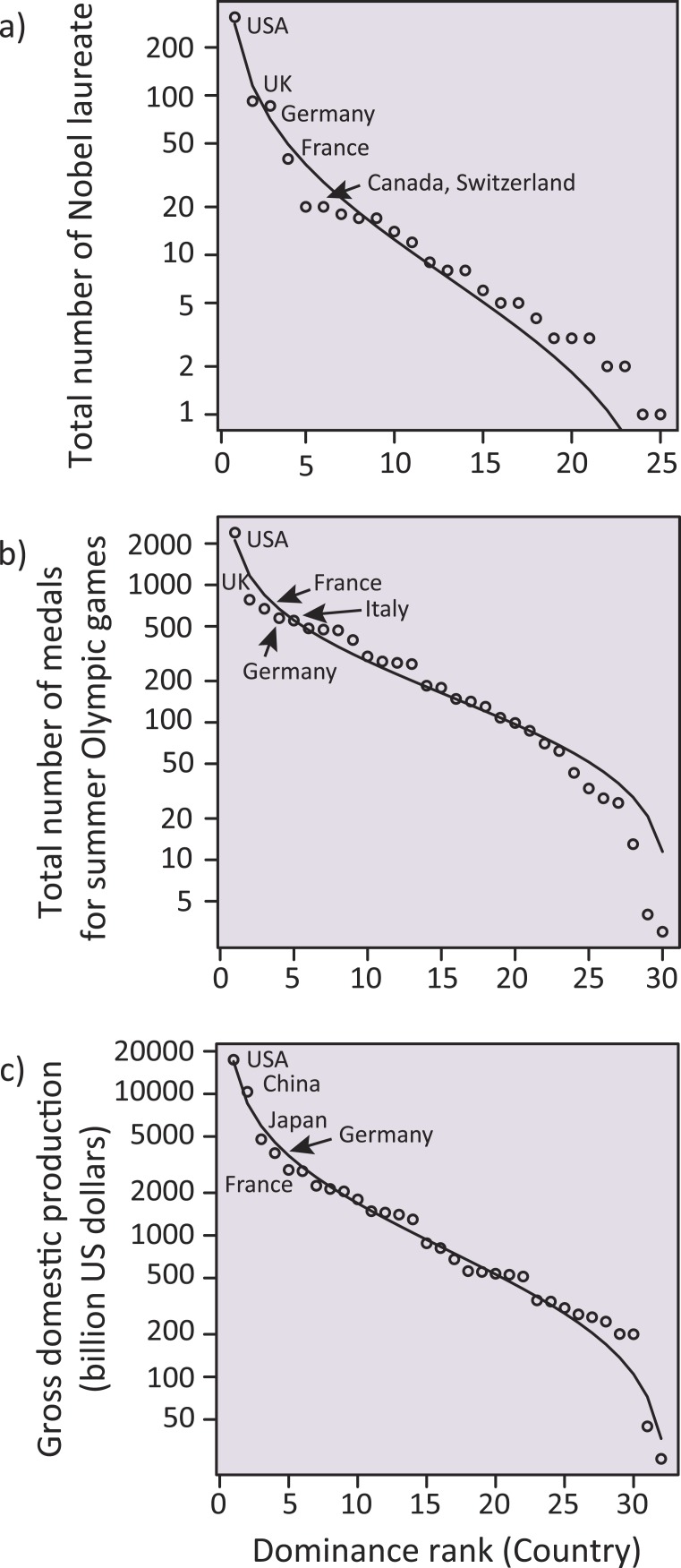
Log-normal distributions analogous to a species-abundance distribution (SAD) for total number of Nobel laureates to 2014, total number of medals for summer Olympic Games to the present (i.e., up to the 2012 London Game), and gross domestic production in 2014 (billion US dollars) for each country. Five dominant countries are labeled within each panel.

Additionally, we investigated whether other societal metrics representing the focal countries in the above analyses followed the SADs (‘Methods’). We found that, consistent with the country-dominance rank for meta-analysis papers, the total number of Nobel laureates, the total number of medals for the summer Olympic Games, and the gross domestic product (GDP) for each country were well-fit to a log-normal distribution (Fig. 5). Nekola & Brown (2007) suggested that, if a system satisfies some conditions inherent in complex systems (such as historical contingency, feedback mechanisms, and system openness, all of which are fundamental to ecosystems), SAD-like patterns could emerge. In addition to such similarity in statistical patterns, we, for the first time, found that the above three country statistics (related to the Nobel laureates, the Olympic medals and the GDPs; Fig. 5) showed a significant positive correlation with the number of ecological meta-analysis papers in each country (Fig. 3) (jackknife regression to remove outlier effects for all rank statistics, all *P* < 0.0001; ‘Methods’). Note that the correlations of the meta-analysis papers with the country statistics for number of Nobel laureates and Olympic medals were not an artifact mediated by GDP (partial correlations that removed the linear effects of GDP, both *r* > 0.71, *P* < 0.0001 and *r* > 0.42, *P* < 0.05 for data including and excluding USA, respectively; ‘Methods’). Taken together, there might be mechanisms generating inequality and hierarchical structure in human society, which are ubiquitous across very different situations (i.e., science, sports, and economy). We speculate that the effects of country reputation on IFs (Table 1) suggests a possible positive feedback; i.e., studies from already-well recognized countries and other peripheral countries may respectively have a higher and a lower likelihood of being treated as “convincing” during review processes, independent of the potential significance of the study, leading to more publications by these dominants.

Unfortunately, our study suggests that the scientific discipline of ecology, which aims to disentangle the underlying mechanisms of biological diversity and to apply the gained knowledge to conserve diversity, is gradually losing diversity itself. Along with the possible influences of country reputation on academic performance (i.e., IFs), the consequences could be potentially enormous. One may think that the dominance in meta-analytical papers merely reflects the similar dominance of the original publications used for meta-analyses. In some minor, peripheral countries, it may be difficult to access to expensive bibliographic databases, which is fundamentally necessary for meta-analyses. Similarly, the paid access to scientific literature could be contributing to scientific inequality (but this inequality could be alternatively reduced by increased publication of quality science in open access journals). Additionally, the number of paper submissions to journals may differ among countries, potentially yielding the country effects on IFs. Full assessments for these issues are not possible; nevertheless, there are several reasons that we are concerned about the inequality in this scientific domain. Note that there are many forms of academic inequality across different disciplines, likely affecting global society beyond the scientific community of each discipline. In the case of ecology, ecologists are now searching for ways of safeguarding biodiversity and ensuring ecosystem services, which are sustained by biodiversity, for the sake of humanity (Cardinale et al., 2012; Naeem, Duffy & Zavaleta, 2012). To achieve this goal, there is a consensus that diverse opinions from different cultures and nations are essential (e.g., see the British Ecological Society’s “Equality and diversity”; www.britishecologicalsociety.org/blog/category/equality-and-diversity/). This is reflected in the formation of working groups in the IPBES, which places importance in having experts well-balanced among different regions/countries. Thus, the possible academic imbalance that we found may contradict the cross-nation harmony that the global society anticipates.

Notably, we stress that, apart from the concordance of countries’ reputations in different societal situations (SADs; Figs. 3 and 5), the consistent order of country-dominance across these situations implies that global society is still being governed by a societal hierarchy. In nature, there are multiple mechanisms that facilitate the coexistence of minor species and thus maintain diversity, such as negative density-dependence among individuals of dominant species (Bagchi et al., 2014). Similarly, scientific communities need rigorous frameworks that facilitate under-represented members, such as double-blinded reviews (Budden et al., 2008) and adequate linguistic editing advice by publishers (Clavero, 2010), although the issue is not limited to disparities among nations (Budden et al., 2008; Ceci & Williams, 2011; Lortie et al., 2007). Note that, in ecological communities (species assemblages), anthropogenic impacts can lead to a stronger dominance of abundant species (Mori et al., 2015), resulting in the further skewedness of SADs (Simons et al., 2014) (i.e., strengthening inequality). From a conservation perspective, even in ecological assemblages that are inherently unequal in terms of dominance distribution among different species, such marked changes and excessive skewedness of SADs are of concern (Matthews, Whittaker & Fuller, 2015; Simons et al., 2014). These latest findings in community ecology also have profound implications for considering inequality issues in human society such as those discussed here. Last, we refer to the notion of Deaton (2014), who stated in a recent commentary that “the rich may write the rules in their favor, and they may work against the public provision of health care or education, for which they pay a large share but have little personal need.” In the light of his words about global sustainability, we hope that the inequality in the discipline of ecology and that in other disciplines will not disproportionally inform society and policy.

## Supplemental Information

10.7717/peerj.1457/supp-1 Table S1 Dataset

10.7717/peerj.1457/supp-2 Table S2 A checklist for the screening protocol of paper selection in this study Note that the present study focused on formal meta-analysis papers (see ‘Methods’) but not conducted a formal analysis based on meta-analytical statistics so that some sections in the below list was not applicable (NA) here. From: Moher D, Liberati A, Tetzlaff J, Altman DG, The PRISMA Group (2009). Preferred Reporting Items for Systematic Reviews and Meta-Analyses: The PRISMA Statement. PLoS Med 6(6): e1000097.

10.7717/peerj.1457/supp-3 Figure S1 A flow diagram of the screening protocol for paper selection in this study From: Moher D, Liberati A, Tetzlaff J, Altman DG, The PRISMA Group (2009). Preferred Reporting Items for Systematic Reviews and Meta-Analyses: The PRISMA Statement. PLoS Med 6(6): e1000097. doi:10.1371/journal.pmed1000097

10.7717/peerj.1457/supp-4 Figure S2 Temporal trend in the number of ecology papers in journals that the present study focused on Data in 2014 is not shown because it has not been yet available in the ISI web of science. Note that multidisciplinary journals were excluded as they included numerous non-ecology papers.

10.7717/peerj.1457/supp-5 Figure S3 Rarefaction curves for the data matrix of first author’s affiliated *country* (column) × *year* (row); (A) individual-based rarefaction for each year in the period of 1998–2014 and (B) sample-based rarefaction to estimate richness change associated with different sample sizes (different number of publication years) Solid curves are rarefaction curves and dashed vertical lines are 95% confidence intervals.

10.7717/peerj.1457/supp-6 Figure S4 Temporal trends in the number of meta-analysis papers in ecology for different study categories; (A) study taxa, (B) practical issues, (C) study systems, and (D) study systems

10.7717/peerj.1457/supp-7 Figure S5 Species abundance distributions (SADs) for meta-analysis papers in ecology The SADs are plotted with a log-normal model for data (A) between 1998 and 2002, (B) between 2003 and 2007 and (C) between 2008 and 2012. The curves were fit with a log-normal model. Five dominant countries were indicated in each panel.

## Additional Information and Declarations

### Data Availability

The following information was supplied regarding data availability: The dataset is provided as Table S1.

## References

[ref-1] Adler PB, Hillerislambers J, Levine JM (2007). A niche for neutrality. Ecology Letters.

[ref-2] Almeida-Neto M, Guimaraes P, Guimaraes PR, Loyola RD, Ulrich W (2008). A consistent metric for nestedness analysis in ecological systems: reconciling concept and measurement. Oikos.

[ref-3] Bagchi R, Gallery RE, Gripenberg S, Gurr SJ, Narayan L, Addis CE, Freckleton RP, Lewis OT (2014). Pathogens and insect herbivores drive rainforest plant diversity and composition. Nature.

[ref-4] Bates D, Maechler M, Bolker B, Walker S (2014). lme4: linear mixed-effects models using Eigen and S4.

[ref-5] Boyd I (2013). Research: a standard for policy-relevant science. Nature.

[ref-6] Budden AE, Tregenza T, Aarssen LW, Koricheva J, Leimu R, Lortie CJ (2008). Double-blind review favours increased representation of female authors. Trends in Ecology and Evolution.

[ref-7] Button K, Nijkamp P (1997). Environmental policy assessment and the usefulness of meta-analysis. Socio-Economic Planning Sciences.

[ref-8] Cardinale BJ, Duffy JE, Gonzalez A, Hooper DU, Perrings C, Venail P, Narwani A, Mace GM, Tilman D, Wardle DA, Kinzig AP, Daily GC, Loreau M, Grace JB, Larigauderie A, Srivastava DS, Naeem S (2012). Biodiversity loss and its impact on humanity. Nature.

[ref-9] Ceci SJ, Williams WM (2011). Understanding current causes of women’s underrepresentation in science. Proceedings of the National Academy of Sciences of the United States of America.

[ref-10] Clavero M (2010). “Awkward wording. Rephrase”: linguistic injustice in ecological journals. Trends in Ecology and Evolution.

[ref-11] Clavero M (2011). Language bias in ecological journals. Frontiers in Ecology and the Environment.

[ref-12] Deaton A (2014). Inevitable inequality?. Science.

[ref-13] Doi H, Mori T (2013). The discovery of species-abundance distribution in an ecological community. Oikos.

[ref-14] Gotelli NJ, Colwell RK (2001). Quantifying biodiversity: procedures and pitfalls in the measurement and comparison of species richness. Ecology Letters.

[ref-15] Henrissat B (1991). National publication bias. Nature.

[ref-16] Jeffery S, Verheijen FGA, Bastos AC, Van Der Velde M (2014). A comment on ‘Biochar and its effects on plant productivity and nutrient cycling: a meta-analysis’: on the importance of accurate reporting in supporting a fast-moving research field with policy implications. GCB Bioenergy.

[ref-17] Kim S (2012). ppcor: partial and Semi-partial (Part) correlation.

[ref-18] Koricheva J, Gurevitch J, Gómez-Aparicio L (2014). Uses and misuses of meta-analysis in plant ecology. Journal of Ecology.

[ref-19] Kunte K (2008). Competition and species diversity: removal of dominant species increases diversity in Costa Rican butterfly communities. Oikos.

[ref-20] Laliberte E, Zemunik G, Turner BL (2014). Environmental filtering explains variation in plant diversity along resource gradients. Science.

[ref-21] Leimu R, Koricheva J (2005). What determines the citation frequency of ecological papers?. Trends in Ecology and Evolution.

[ref-22] Lortie CJ, Aarssen LW, Budden AE, Koricheva JK, Leimu R, Tregenza T (2007). Publication bias and merit in ecology. Oikos.

[ref-23] Magurran AE, Henderson PA (2003). Explaining the excess of rare species in natural species abundance distributions. Nature.

[ref-24] Magurran AE, McGill BJ (2011). Biological diversity.

[ref-25] Matthews TJ, Borges PAV, Whittaker RJ (2014). Multimodal species abundance distributions: a deconstruction approach reveals the processes behind the pattern. Oikos.

[ref-26] Matthews TJ, Whittaker RJ, Fuller R (2015). On the species abundance distribution in applied ecology and biodiversity management. Journal of Applied Ecology.

[ref-27] McGill BJ (2010). Towards a unification of unified theories of biodiversity. Ecology Letters.

[ref-28] McGill BJ, Etienne RS, Gray JS, Alonso D, Anderson MJ, Benecha HK, Dornelas M, Enquist BJ, Green JL, He F, Hurlbert AH, Magurran AE, Marquet PA, Maurer BA, Ostling A, Soykan CU, Ugland KI, White EP (2007). Species abundance distributions: moving beyond single prediction theories to integration within an ecological framework. Ecology Letters.

[ref-29] Mechanic D (2002). Disadvantage, inequality, and social policy. Health Affairs.

[ref-30] Mori AS, Ota AT, Fujii S, Seino T, Kabeya D, Okamoto T, Ito MT, Kaneko N, Hasegawa M (2015). Biotic homogenization and differentiation of soil faunal communities in the production forest landscape: taxonomic and functional perspectives. Oecologia.

[ref-31] Naeem S, Duffy JE, Zavaleta E (2012). The functions of biological diversity in an age of extinction. Science.

[ref-32] Nekola JC, Brown JH (2007). The wealth of species: ecological communities, complex systems and the legacy of Frank Preston. Ecology Letters.

[ref-33] Oksanen J, Blanchet FG, Kindt R, Legendre P, Minchin PR, O’Hara RB, Simpson GL, Solymos P, Stevens MHH, Wagner H (2013). vegan: community ecology package.

[ref-34] Osterloh M, Frey BS (2015). Ranking games. Evaluation Review.

[ref-35] Perrings C, Duraiappah A, Larigauderie A, Mooney H (2011a). The biodiversity and ecosystem services science-policy interface. Science.

[ref-36] Perrings C, Naeem S, Ahrestani FS, Bunker DE, Burkill P, Canziani G, Elmqvist T, Fuhrman JA, Jaksic FM, Kawabata ZI, Kinzig A, Mace GM, Mooney H, Prieur-Richard A-H, Tschirhart J, Weisser W (2011b). Ecosystem services, targets, and indicators for the conservation and sustainable use of biodiversity. Frontiers in Ecology and the Environment.

[ref-37] R Core Team (2013). R: a language and environment for statistical computing.

[ref-38] Rader R, Bartomeus I, Tylianakis JM, Laliberté E, Van Kleunen M (2014). The winners and losers of land use intensification: pollinator community disassembly is non-random and alters functional diversity. Diversity and Distributions.

[ref-39] Ravallion M (2014). Income inequality in the developing world. Science.

[ref-40] Sasaki T, Katabuchi M, Kamiyama C, Shimazaki M, Nakashizuka T, Hikosaka K (2012). Nestedness and niche-based species loss in moorland plant communities. Oikos.

[ref-41] Simons NK, Gossner MM, Lewinsohn TM, Lange M, Turke M, Weisser WW (2014). Effects of land-use intensity on arthropod species abundance distributions in grasslands. Journal of Animal Ecology.

[ref-42] Šímová I, Storch D, Keil P, Boyle B, Phillips OL, Enquist BJ (2011). Global species–energy relationship in forest plots: role of abundance, temperature and species climatic tolerances. Global Ecology and Biogeography.

[ref-43] Tukey JW (1958). Bias and confidence in not-quite large samples. Annals of Mathematical Sciences.

[ref-44] Turnhout E, Bloomfield B, Hulme M, Vogel J, Wynne B (2012). Conservation policy: listen to the voices of experience. Nature.

[ref-45] Ulrich W, Ollik M, Ugland KI (2010). A meta-analysis of species-abundance distributions. Oikos.

