# Supplementary material for: Academic inequality through the lens of community ecology: a meta-analysis
Source: PeerJ. 2015 Dec 3;3:e1457. doi: 10.7717/peerj.1457 (PMC4671160; doi:10.7717/peerj.1457)
Supplement: Figure S1 — From: Moher D, Liberati A, Tetzlaff J, Altman DG, The PRISMA Group (2009). Preferred Reporting Items for Systematic Reviews and Meta-Analyses: The PRISMA Statement. PLoS Med 6(6): e1000097. doi:10.1371/journal.pmed1000097 [file peerj-03-1457-s003.doc]

**Screening**

**Included**

**Eligibility**

**Identification**

Records identified through database searching
(n = 456)

Additional records identified through other sources
(n = 448)

Records after duplicates removed
(n = 904)

Records screened
(n = 454)

Records excluded
(n = 450)

Full-text articles assessed for eligibility
(n = 454)

Full-text articles excluded, with reasons
(n = 0)

Studies included in qualitative synthesis
(n = 454)

Studies included in quantitative synthesis (meta-analysis)
(n = 454)
